# Algaecidal and oxidative effects of metal-free phthalocyanine beta tetra-substituted with sodium 2-mercaptoethanesulfonate

**DOI:** 10.55730/1300-0527.3313

**Published:** 2021-11-22

**Authors:** Hatice TUNCA, Sena ÇAĞATAY ÖZPINAR, Ayşegül TEKBABA, Tuğba ONGUN SEVİNDİK, Ali DOĞRU, Armağan GÜNSEL, Ahmet T. BİLGİÇLİ, M. Nilüfer YARAŞIR

**Affiliations:** 1 Department of Biology, Faculty of Arts and Sciences, Sakarya University, Sakarya, Turkey; 2 Department of Chemistry, Faculty of Arts and Sciences, Sakarya University, Sakarya, Turkey

**Keywords:** Microalgae, sulfonated phthalocyanine, antioxidant, oxidative stress, growth rate

## Abstract

In this study, a water-soluble metal-free phthalocyanine (SPC) containing sodium 2-mercaptoethanesulfonate substituents at the peripheral positions was used to investigate the algaecidal properties and oxidative effects on the growth of two microalgal species, *Arthrospira platensis* and *Chlorella vulgaris*. Although OD at 560 nm and chlorophyll-*a* content were decreased in *Arthrospira platensis* during 7 days depending on dose and time, increases in both OD at 750 and chlorophyll-*a* content at 8 ppb (parts per billion) concentration on the 7th day were observed in *Chlorella vulgaris*. However, total SOD (superoxide dismutase) and GR (glutathione reductase) enzyme activity of *A. platensis* cultures did not display any alteration in all concentrations, SOD activity displayed an increase significantly at 2 ppb concentration, and GR activity showed increases at 1, 2, and 4 ppb concentrations in *C. vulgaris* application. In *A. platensis* application, APX (ascorbate peroxidase) activity decreased at 0.50 ppb, 1 ppb, and 1.5 ppb concentrations. In addition, *C. vulgaris* application showed decreases at all concentrations. When MDA content increased at all concentrations, the H_2_O_2_ content increased only at significatly 0.125 ppb concentration in *A. platensis* cultures. Both MDA (malondialdehyde) and H_2_O_2_ (hydrogen peroxide) content of *C. vulgaris* cultures showed a statistically significant decrease at all concentrations compared to control. Free proline decreased at 0.25 ppb, 0.50 ppb, 1 ppb, and 1.5 ppb concentrations in *A. platensis* application, and it decreased at all the concentrations of *C. vulgaris* application. It concluded that this compound has inhibition effects on *A. platensis*, but it supports growth in *C. vulgaris*. Therefore, this synthesized phthalocyanine compound (SPC) should be consumed carefully, and the contamination to aquatic ecosystems should be prevented.

## 1. Introduction

Phytoplankton is the general name given to organisms that have adapted to suspend in the pelagic water of aquatic environments such as sea, lake, pond, and river [1]. Planktonic primary producers form the basis of the food chain in the aquatic environments and accumulate the xenobiotics within their cells, and, thus, these compounds reach humans through this network. It also has an important role in producing 50%–90% of atmospheric oxygen in the world, and a change in phytoplankton affects the community composition of the entire aquatic ecosystem [2].

*A. platensis* is naturally found in lakes in sub-tropical areas with high alkalinity, but it is a cosmopolitan organism that can live in a wide variety of aquatic environments such as turbid stagnant waters, streams, fresh and brackish waters. It has a spiral filament type that can also be found in straight strains. It is a prokaryotic cyanobacterium, and it has commercial importance [3]. *C. vulgaris* is a common eukaryotic microalga that lives in freshwaters. Besides, they can form green covers on bark of trees and stones. *C. vulgaris* cells are spherical, and the coccoid type and their sizes vary from 5 μm to 10 μm. The thickness of the cell wall varies according to the growth phase and basically it protects the cell from stimulants in the external environment and pests [4–5].

Phthalocyanines (Pcs) are like porphyrins, but they are tetropyrotic macromolecules separated from them by nitrogen atoms bound to pyrrole units. The addition of benzene rings to macrocycles enhances absorbance at longer wavelengths than porphyrins [6] and allows the use of phthalocyanines in various disciplines [7]. These substances have also been used as antibacterial, antifungal, and antitumoral in photodynamic therapy in various studies [8–10]. Drabkova et al. [11] and Jančula et al. [7] reported that these compounds have adverse effects against microalgae. These valuable properties of Pcs are due to the cytotoxic effects of produced single oxygen and reactive oxygen species, (ROS) [10]. Pcs can lead to the formation of singlet oxygen (_1_O^2^) in the presence of visible light and diatomic oxygen. Singlet oxygen (_1_O^2^) has obstructive effects on growth rate and metabolic activities. These molecules oxidize biological structures and, thus, destroy cells [12]. The amount of produced singlet oxygen and photocytotoxicity of phthalocyanines and water-soluble sulfonated derivatives vary according to the degree of sulfonation. The addition of sulfonate groups around the Pcs greatly increases the solubility of these compounds and eliminates the need for liposomal delivery vehicles [6].

In previous studies, it has been demonstrated that singlet oxygen species have an inhibitory effect via binding to the negatively charged cell membranes of various algae [13]. Even if there is no significant environmental stress, these molecules can occur in algae, cyanobacterial cells, and plants as a result of metabolism, but they can prepare cells for oxidative stress by functioning as signal molecules if their amount increases [14, 15]. In this case, it can be toxically harmful in cells, by mainly causing degradation of nucleic acids, lipids, and proteins [15]. Therefore, their occurrence is often well protected by certain mechanisms in cells. These mechanisms are known as antioxidants and the most important these enzymatic mechanisms are superoxide dismutase (SOD), glutathione reductase (GR), and ascorbate peroxidase (APX) that are correlated with each other [16]. SOD is an enzyme that reduces the superoxide content by converting superoxide radicals into water and hydrogen peroxide [17,18]. APX converts hydrogen peroxide into dehydroascorbate and water by using ascorbic acid. GR reduces oxidized glutathione into reduced glutathione to reproduce ascorbic acid and to maintain the activity of APX in cells [19–23]. In non-enzymatic parameters, proline is known to increase during the oxidative stress process [24–25]. On the other hand, MDA is a molecule that provides the evaluation of membrane damage in cells and is formed by the oxidation of fatty acids containing more than one double bond [26–27].

Although there are some studies in the literature on the effects of pesticides and heavy metals on algae, there is no study on phthalocyanines.

Although there have been many studies related to oxidative damage of pesticides, heavy metals, and other chemicals on microalgae, there have been no studies about impact of water contaminated with phthalocyanines containing sulfonate groups on phytoplanktonare in the literature. Since the most important difference between a medicine and a poison is the concentration to which it is applied, the application concentrations of each newly produced compound should be determined. Therefore, the aims of this present study are (i) to investigate whether the excessive usage of the synthesized phthalocyanine compound (SPC) has a negative effect on algae and (ii) to evaluate the suitability of the synthesized phthalocyanine compound (SPC) as an algaecide with its adequate dosage to prevent algal blooms occurring in the lake ecosystems.

## 2. Material and methods

### 2.1. Algae culture and treatment

Soley Microalgae Institute (California, USA) (Culture collection No: SLSP01) and Çukurova University supplied the cultures of *A. platensis*-M2 and *C. vulgaris*, and the aliquots were cultivated in Spirulina Medium [28] and BG11 [29] under axenic conditions, respectively. The 200 mL of *A. platensis* and *C. vulgaris* pre-cultures growed at 30±1 °C and 25±1 °C, respectively. The mimicry conditions of circadian rhythm are 12:12 h, at 6800 lux, during the seven days. At the end of this period, cultures were inoculated as 50 mL of algal culture. 2(3), 9(10), 16(17), 23(24)-Tetrakis-(sodium 2-mercaptoethanesulfonate) metal-free phthalocyanine compound (SPC) was synthesized according to the procedure of Günsel et al. [30] (Scheme). Various concentrations of the synthesized phthalocyanine compound (SPC) (0–1.5 ppb for *A. platensis* and 0–8 ppb for *C. vulgaris*) were dripped to the cultures. EC50 value was used for the determination of concentrations ranges in preliminary bioassays.

### 2.2. Cell growth and chlorophyll-*a* assay

Optical density (OD) of control and stress-treated cultures were determined spectrophotometrically during the 7 days. OD at 560 nm was preferred for *A. platensis*, and OD at 750 nm was selected for *C. vulgaris* to adjust the most appropriate growth plots. Chlorophyll-*a* extracts were obtained by methanol, and the contents were determined by spectrophotometrical methods during the 7 days [31].

### 2.3. Antioxidant enzyme activities

The culture extractions were conducted according to Tunca et al. [25], and the samples were deep-freezed until enzyme assays (at −20 °C). On the assay day, samples were pounded with liquid nitrogen and specific buffers of each enzyme were used to suspend the samples. Bradford’s [32] method was carried out to measure the protein concentrations of microalgae yield. In the assay, bovine serum albumin (BSA) was used as a standard.

Beyer and Fridovich [33] method was modified to determine the SOD activity and NBT (nitroblue tetrazolium) photoreduction principle were utilized in this assay.1.5 mL buffer was prepared with 100 mM K_2_HPO_4_ buffer (pH 7.0), 1 mM Na_2_EDTA and 2% PVP for homogenization. The samples were centrifugated at 14.000 rpm and 4 °C for 20 min. After treatment, SOD activity was measured as described in Önem et al. [34]. The standard graphic was prepared, and it was used to calculate the quantity of SOD activity as unit mg^−1^ protein.

Wang et al [35] method was modified to determine the APX activity. The estimation of the ascorbate oxidation decreasing rate at 290 nm was utilized as principle. Önem et al. [34] were perfomed for extraction and determination of the enzyme activity. The initial rate of the reaction using the extinction coefficient of ascorbate (E = 2.8 mM cm^−1^ at 290 nm) was utilized to calculate enzyme activity.

Sgherri et al. [36] method was modifed to measure the GR activity. 1.5 mL buffer was prepared with 100 mM K_2_HPO_4_ buffer (pH 7.0), 1 mM Na_2_EDTA and 2% PVP for homogenization. The GR activity was measured as described in Önem et al. [34]. The initial rate of the reaction after subtracting the non-enzymatic oxidation using the extinction coefficient of NADPH (E = 6.2 mM cm^−1^ at 340 nm) was utilized to calculate the enzyme activity.

### 2.4. Determination of malondialdehyde and hydrogen peroxide

Heath and Packer [37] method was modified to determine the MDA content. A total of 3 mL of 0.1% TCA (Trichloroacetic acid) (4 °C) was used for homogenization of 0.2 g pellet. After centrifugation at 4100 rpm for 15 min, the supernatant was used to perform the assay as described in Tunca et al. [25]. The non-specific absorbance at 600 nm and the absorbance at 532 nm were recorded, and they substracted from each other. The extinction coefficient of 155 mM^−1^ cm^−1^ was used to determine the MDA content. 1 mL of 1 M KI, 0.5 mL of 0.1 M Tris–HCl (pH = 7.6), and 0.5 mL of supernatant were mixed to determinate of the H_2_O_2_ content. After 90 min, the absorbance at 390 nm was measured, and H_2_O_2_ content was calculated as described in Tunca et al. [25].

### 2.5. The proline content determination

Weimberg et al.’s [38] method was modified to determine the proline content. A total of 10 Ml of 3% aqueous sulphosalicylic acid was used to homogenize 0.1 g of pellet. The samples were used to carry out as described in Tunca et al. [25], and the absorbance at 520 nm was measured by spectrophotometrical methods.

### 2.6. Statistical analysis

The differences between the control and treated samples were analyzed by one-way ANOVA, with a 95% confidence interval according to LSD. Three biological replicate cultures were used for each treatment. The mean values ± SE were given in Figures.

## 3. Results

**Figure 1:**
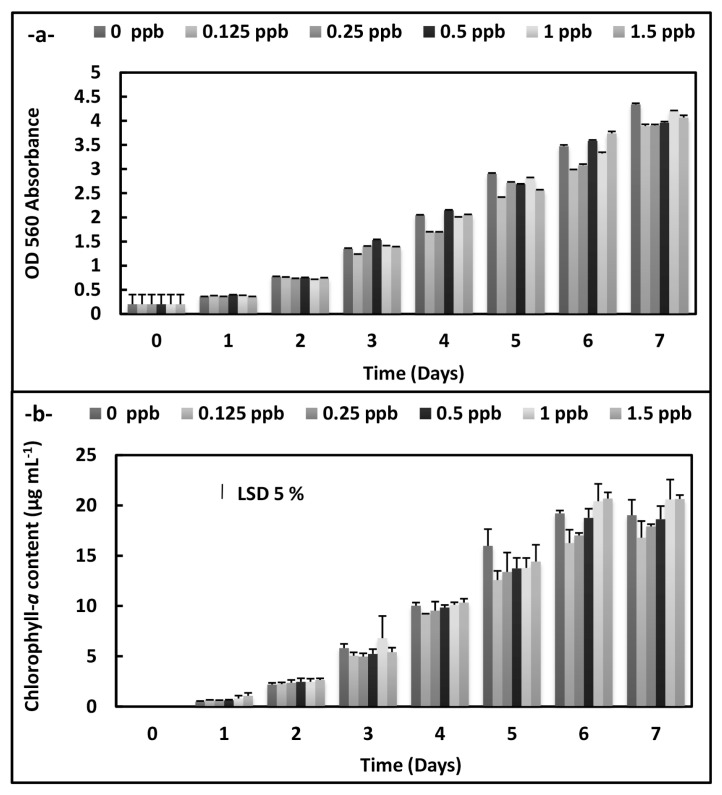
Biomass values and chlorophyll-*a* values of *A. platensis* supplemented with the phthalocyanine compound (SPC) (0–1.5 ppb) during the 7 days. Data are the means ± SE of three replicates.

**Figure 2:**
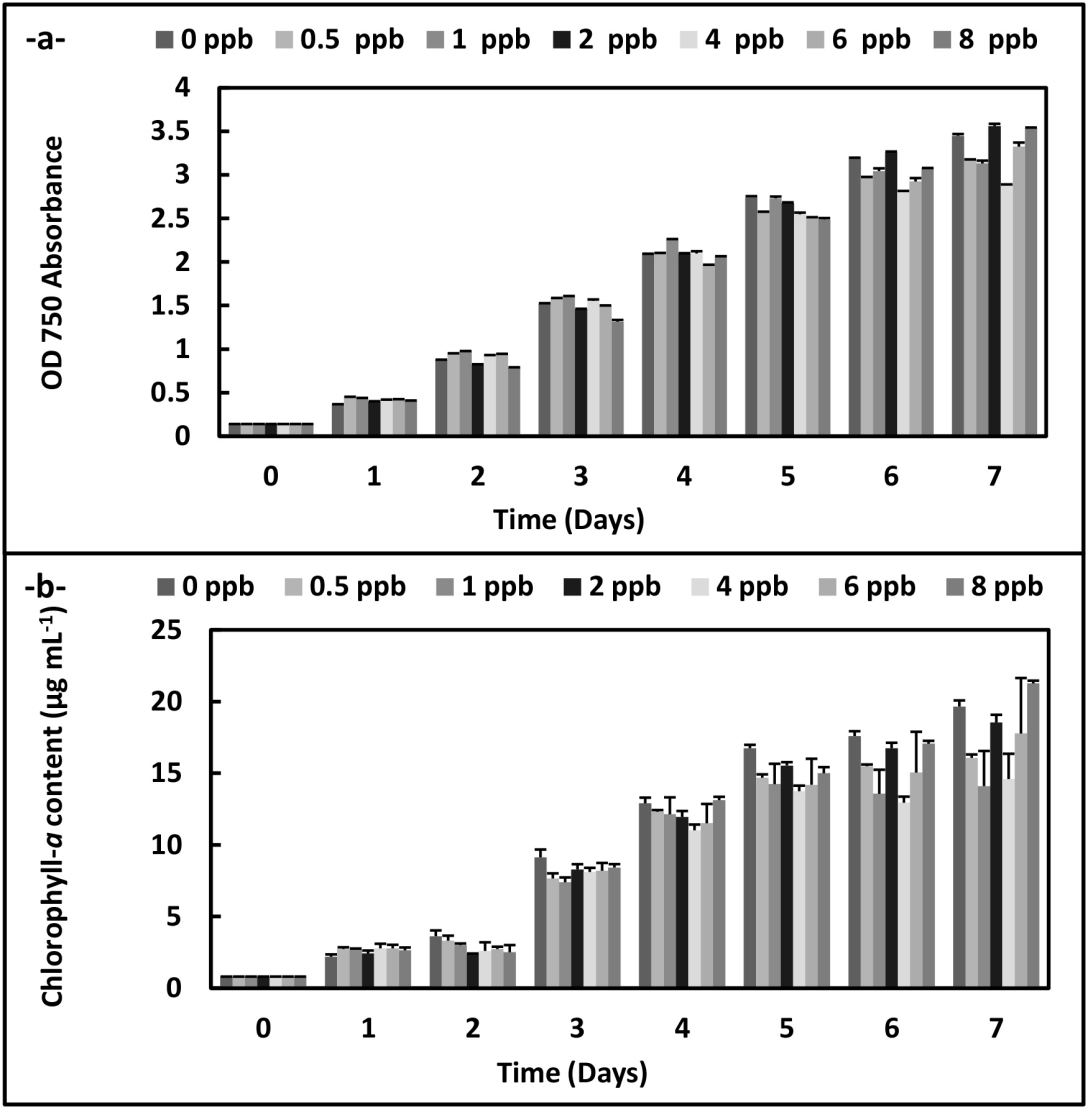
Biomass values and chlorophyll-*a* values of *C. vulgaris* supplemented with the phthalocyanine compound (SPC) (0–8 ppb) during the 7 days. Data are the means ± SE of three replicates.

There were statistically significant decreases at OD 560 absorbance in *A. platensis* cultures according to an increase of the phthalocyanine (SPC) concentrations during the 7 days (p < 0.05) (Figure 1). On the other hand, the increases were observed on the growth plot obtained from OD 750 absorbance at 8 ppb concentration on the 7 th day in *C. vulgaris* cultures exposed to the phthalocyanine compound (SPC). Similarly, chlorophyll-*a* content rises at the same conditions (p < 0.05) (Figure 2).

**Figure 3:**
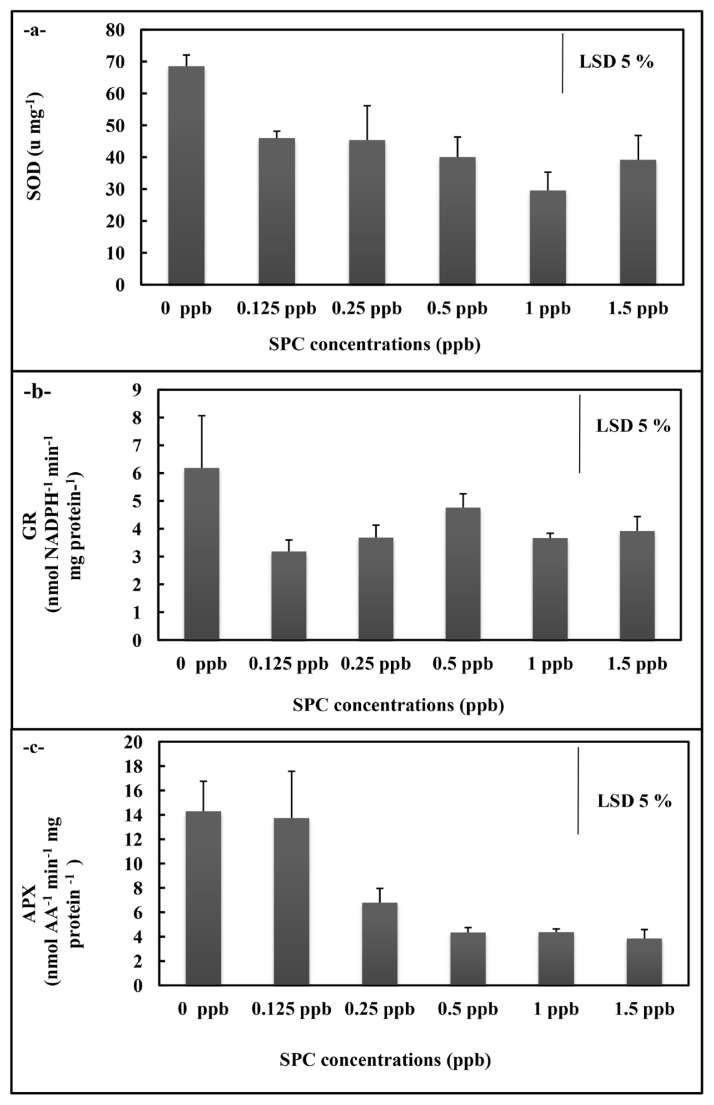
Total SOD (a), GR (b) and APX (c) activities of *A. platensis* supplemented with the phthalocyanine compound (SPC) (0–1.5 ppb). Data are the means ± SE of three replicates.

**Figure 4:**
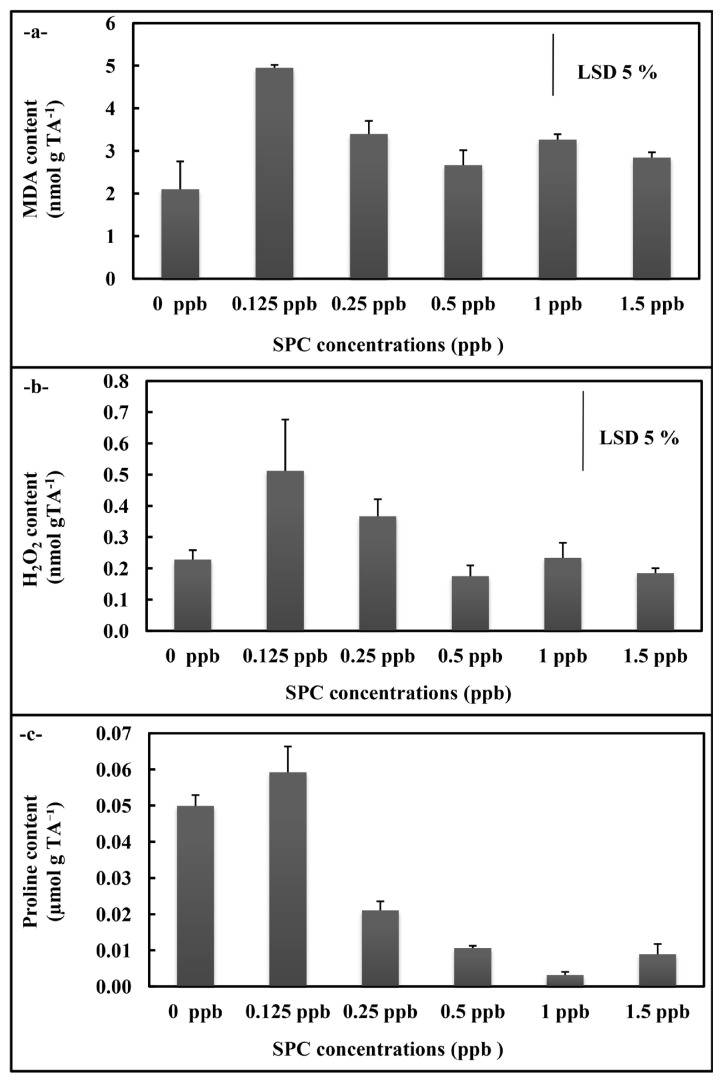
MDA (a), H_2_O_2_ (b) and proline (c) contents of *A. platensis* supplemented with the phthalocyanine compound (SPC) (0–1.5 ppb). Data are the means ± SE of three replicates.

In *A. platensis* application, the total SOD and GR activities of *A. platensis* cultures did not display significant alteration in the overall concentrations (Figure 3a, 3b), but APX activity decreased at 0.25, 0.50 ppb, 1 ppb, and 1.5 ppb concentrations compared to control (p < 0.05) (Figure 3c). While the MDA content rised at all concentrations (p < 0.05) (Figure 4a), the H_2_O_2_ content statistically significantly raised at 0.125 ppb concentration (p < 0.05)(Figure 4b). The free proline content displayed decreases at 0.25 ppb, 0.50 ppb, 1 ppb, and 1.5 ppb concentrations (p < 0.05) (Figure 4c).

**Figure 5:**
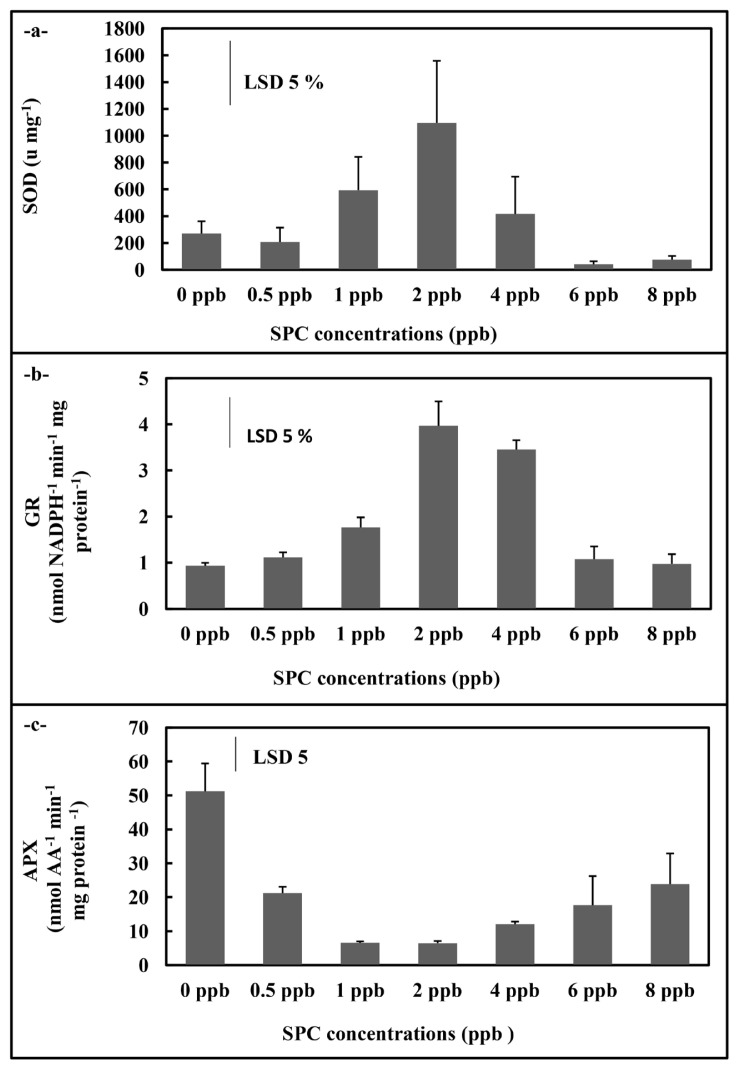
Total SOD (a), GR (b) and APX (c) activities of *C. vulgaris* supplemented with the phthalocyanine compound (SPC) (0–8 ppb). Data are the means ± SE of three replicates.

**Figure 6:**
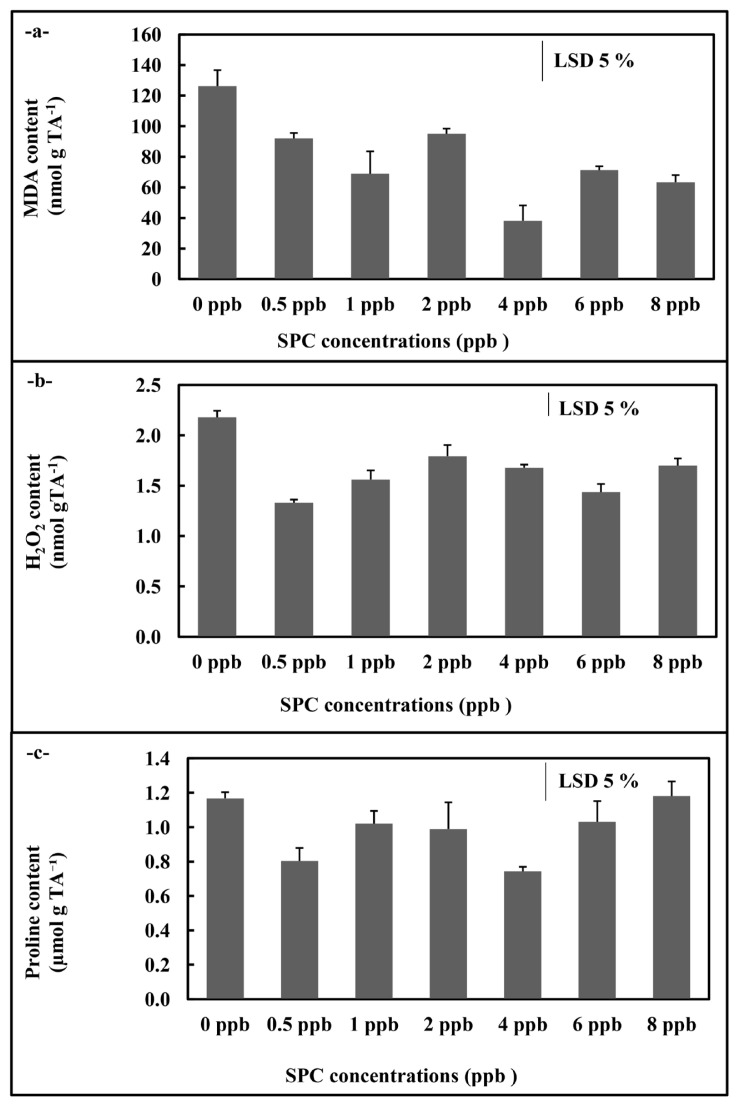
MDA (a), H_2_O_2_ (b) and proline (c) contents of *C. vulgaris* supplemented 0–8 ppb with the phthalocyanine compound (SPC). Data are the means ± SE of three replicates

In *C. vulgaris* application, SOD activity showed an increase at 2 ppb concentration compared to control (p < 0.05) (Figure 5a). Also, the GR activity displayed increases compared to control at 1, 2, and 4 ppb concentrations (p < 0.05) (Figure 5b). On the contrary, the APX activity showed a significant decrease compared to control at all concentrations (p < 0.05)(Figure 5c). Moreover, both MDA and H_2_O_2_ content of *C. vulgaris* cultures showed significant decreases at all concentrations compared to control (p < 0.05) (Figures 6a,6b). The free proline amount displayed decreases at concentrations (0.5 and 4 ppb) of the phthalocyanine compound (SPC) (Figure 6c).

## 4. Discussion

In this study, the effects of the synthesized phthalocyanine compound (SPC) were investigated in terms of some parameters such as the absorbance of OD560 and OD750, the contents of chlorophyll-*a*, H_2_O_2_, malondialdehyde, and proline, and the activities of SOD, APX, and GR in *A. platensis* and *C. vulgaris*.

It can be concluded that there is a decrease in the growth rate of *A. platensis* with the phthalocyanine compound (SPC) application, but interestingly, an increase was observed in *C. vulgaris* application at 8 ppb concentration especially on the 7th day. As a result of its enhancer effect on *C. vulgaris* growth, commercial use of the synthesized phthalocyanine compound (SPC) for *C. vulgaris* cultures may be considered.

Morelli et al. [39] reported that APX and GR activities did not significantly change, even at the higher water-soluble CdSe quantum dots (QD) concentrations of water-soluble QDs in *Phaeodactylum tricornutum*. This situation may be explained as the level of reduced glutathione is a strong free radical scavenger. Also, in our study, the absence of reduced glutathione in the glutathione pool may be related to the decreased APX activity. It was observed that SOD and GR activity did not change, but only APX activity significantly decreased in *A. platensis* with the phthalocyanine compound (SPC) application. Günsel et al. [40] observed that while the antioxidant SOD enzyme activity decreased at the 0–15 μg mL^−1^ concentration with the application of water-soluble phthalocyanine derivative, while the APX and GR activities did not change statistically. It can be concluded that phthalocyanine derivatives show different oxidative stress characteristics depending on their content. Phthalocyanine tetrasulfonate tetra sodium has generating capacity of ROS much than other phthalocyanines, so the compound in the study was more toxic than the one used in Günsel et al. [40]. Tekbaba et al. [41] found that SOD activity increased, while the GR activity decreased with the application of water-soluble copper phthalocyanine containing sulfonate groups in *A. platensis*, but it did not change the APX content at similar concentrations to our study. This significant difference indicates that the metal bonded groups in phthalocyanines significantly alter the free oxygen generating capacity of the compound.

Fang et al. [42] reported that 4,4′-di-CDPS and 4,4′-di-CDE chemicals inhibited the activity of antioxidant enzymes, and, thus, the chemicals increased to MDA content of *Scenedesmus obliquus*. Likewise, deficiency of these three key enzymes in oxidative stress response is related to the increase of MDA at all concentrations and H_2_O_2_ content at 0.125 ppb in our study, and the H_2_O_2_ content may not have changed at high concentrations due to the activities of oxidases such as amino acid oxidase glucose oxidase, glycolate oxidase, and sulfite oxidase [43].

Ding et al. [44] observed that SOD activity increased at low naproxen (NPX) concentration (1 mg L^−1^), but it decreased at higher concentrations (at 50 and 100 mg L^−1^) by NPX application on *Cymbella* sp. and *Scendesmus quadricauda*. They explained that higher NPX levels caused the accumulation of OH^−^ and H_2_O_2_ in algal cells. On the other hand, Melegari et al. [45] observed that MDA content did not change by increasing antioxidant enzyme activities in *Chlamydomonas reinhardtii*. According to Tekbaba et al. [41], water-soluble copper phthalocyanine containing sulfonate groups increased the SOD and GR activites, but it decreased the APX activity. In our study, SOD and GR enzyme activities increased at low doses but did not show any effect at high doses, possibly due to enzyme degradation caused by the synthesized phthalocyanine compound (SPC) in *C. vulgaris* application.

Tekbaba et al. [41] reported that MDA and H_2_O_2_ concentrations in *A. platensis* decreased with water-soluble copper phthalocyanine containing sulfonate groups. In *C. vulgaris* application, these parameters increased. Also, this situation differs from the findings in this study. The use of metal-free phthalocyanine derivatives may provide more growth-promoting effect than water-soluble copper phthalocyanine containing sulfonate groups, and it caused less oxidative damage than metal-containing derivatives in *C. vulgaris*, so it may be more suitable for commercial use.

Radic et al. [46] reported a high negative correlation between photosynthetic pigments and MDA. In our study, the chlorophyll-*a* increased at higher concentration. This explained the decrease of MDA in these concentrations. Also, the proline content may have decreased due to the free radicals formed, in both applications.

In conclusion, this compound caused less oxidative stress in *C. vulgaris* than in *A. platensis* because the eukaryotic cell structure of *C. vulgaris* has a more complex detoxification mechanism. The antioxidant molecules of *A. platensis* was not adequate for the inactivation of the synthesized phthalocyanine compound (SPC), and it caused lipid peroxidation and increased H_2_O_2_ content. However, *C. vulgaris* could respond by its antioxidant system, and the increase of free radicals was prevented. It can be said that this synthesized phthalocyanine compound (SPC) can be used as an algaecidal at the aforementioned concentrations for *A. platensis* and *C. vulgaris* organisms. In addition, the compound can be used for growth-stimulation in commercial cultures of *C. vulgaris*.

## Figures and Tables

**Scheme:**
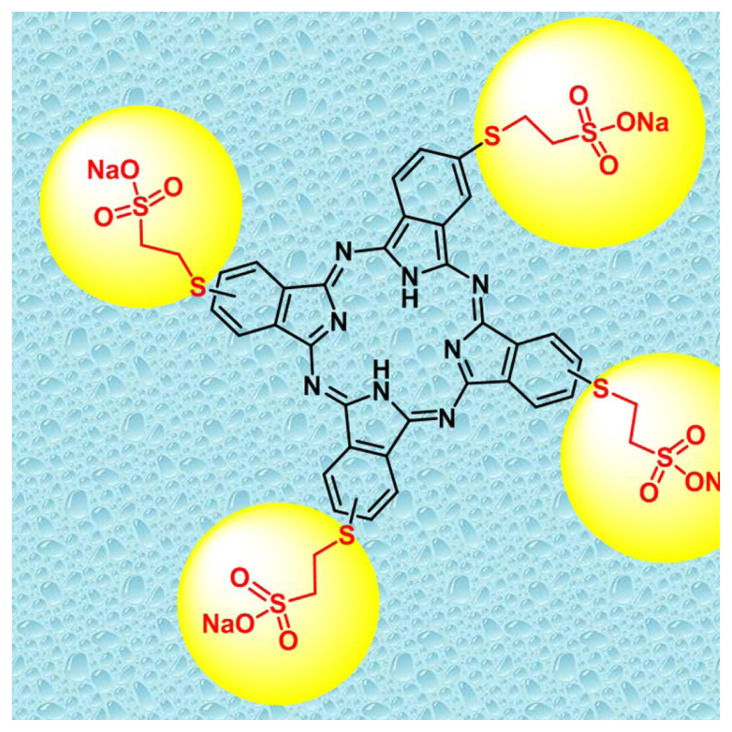
The molecular structure of 2(3), 9(10), 16(17), 23(24)-tetrakis-(sodium 2-mercaptoethanesulfonate) metal-free phthalocyanine compound (SPC).
